# Quantitative modelling of legume root nodule primordium induction by a diffusive signal of epidermal origin that inhibits auxin efflux

**DOI:** 10.1186/s12870-016-0935-9

**Published:** 2016-11-15

**Authors:** Eva E. Deinum, Wouter Kohlen, René Geurts

**Affiliations:** 1 Mathematical and Statistical methods group, Wageningen University, Droevendaalsesteeg 1PB, Wageningen, 6708 the Netherlands; 2 Laboratory for Molecular Biology, Wageningen University, Droevendaalsesteeg 1, Wageningen, 6708 PB the Netherlands; 3 FOM institute AMOLF, Science Park 104XG, Amsterdam, 1098 the Netherlands

**Keywords:** Root nodule formation, Hormone interaction, Developmental signaling, Legume-rhizobium symbiosis

## Abstract

**Background:**

Rhizobium nitrogen fixation in legumes takes place in specialized organs called root nodules. The initiation of these symbiotic organs has two important components. First, symbiotic rhizobium bacteria are recognized at the epidermis through specific bacterially secreted lipo-chitooligosaccharides (LCOs). Second, signaling processes culminate in the formation of a local auxin maximum marking the site of cell divisions. Both processes are spatially separated. This separation is most pronounced in legumes forming indeterminate nodules, such as model organism *Medicago truncatula*, in which the nodule primordium is formed from pericycle to most inner cortical cell layers.

**Results:**

We used computer simulations of a simplified root of a legume that can form indeterminate nodules. A diffusive signal that inhibits auxin transport is produced in the epidermis, the site of rhizobium contact. In our model, all cells have the same response characteristics to the diffusive signal. Nevertheless, we observed the fastest and strongest auxin accumulation in the pericycle and inner cortex. The location of these auxin maxima correlates with the first dividing cells of future nodule primordia in *M. truncatula*. The model also predicts a transient reduction of the vascular auxin concentration rootward of the induction site as is experimentally observed. We use our model to investigate how competition for the vascular auxin source could contribute to the regulation of nodule number and spacing.

**Conclusion:**

Our simulations show that the diffusive signal may invoke the strongest auxin accumulation response in the inner root layers, although the signal itself is strongest close to its production site.

**Electronic supplementary material:**

The online version of this article (doi:10.1186/s12870-016-0935-9) contains supplementary material, which is available to authorized users.

## Background

Nitrogen-fixing endosymbioses require formation of genuine organs to host the diazotrophic microbial partner [1]. The best studied diazotrophic endosymbiosis is the interaction between legume plants and a paraphyletic group of alpha- and beta-proteobacteria collectively known as rhizobium. Rhizobium bacteria trigger the formation of root – and in some cases – stem nodules. These nodules originate from nodule primordia, which are initiated upon rhizobium contact.

Nodule formation is induced upon perception of rhizobial signal molecules: lipo-chitooligosaccharides (LCOs). This causes a group of cortical cells to re-enter the cell cycle [2]. From these cells, a nodule primordium is formed. The potency of cells to divide upon rhizobium LCO signalling is delineated by several constraints. Along the vertical root axis only cortical cells in the late elongation and early differentiation zones are receptive, whereas the radial position is constrained by the organization of the stele. Generally, cells opposite xyleme poles are more receptive than those opposite phloem poles of the vasculature [3, 4]. It was shown in pea (*Pisum sativum*) that this positioning effect depends on ethylene biosynthesis, particularly at the phloem poles, which negatively affects root nodule formation [5]. It was also found that the *Medicago truncatula* (Medicago) ethylene signalling mutant *Mtein2/Mtskl* is less constrained in radial positioning [6–8]. This suggests that local ethylene biosynthesis and subsequent signalling is an important constraint in receptiveness of root cortical cells to divide in response to rhizobial LCO-induced signalling.

Additional variation in axial positioning of nodule primordia in the root cortex is observed between different legume species. In most legumes, nodules originate from inner cortical cells, whereas in a few legume lineages –e.g., Dalbergioid (e.g. lupin), Millettioid (e.g. soybean) and Loteae (e.g. *Lotus*) clades– nodule primordia are formed in more outer cortical cell layers [9]. The initial positioning of the primordium correlates with the growth character of the nodule; determinate or indeterminate, respectively. Indeterminate nodules, which originate from inner cortical cell layers, maintain a persistent meristem at the apex, allowing for sustained nodule growth. In contrast, in the nodules formed by legume species of the Dalbergioid, Millettioid and Loteae clades, meristematic activity ceases upon maturation, which results in a determinate growth phenotype.

A single plant root can carry multiple nodules. Neighbouring nodules typically are well separated. This is true even upon constitutive expression of dominant active alleles of *Calclium/Calmodulin-dependent kinase (CCaMK)* or the cytokinin histidine kinase *LjLHK1/MtCRE1*, both causing a spontaneous nodulation phenotype in Medicago and *Lotus japonicus* (Lotus) in absence of rhizobium [10–13]. Despite their spontaneous initiation, however, also these (pseudo)nodules occur well separated from each other. This suggests the presence of a lateral inhibition mechanism that is automatically activated during nodule primordium development. The underlying molecular mechanism of this lateral inhibition has not yet been uncovered. Some insights, however, have been obtained by genetic studies. It was found that that *ein2* mutants in Lotus and Medicago do not form large, well separated nodules, but rather form numerous small nodules joined in one or more clusters that have a certain spacing on the root [6–8, 14, 15]. This suggests that inhibitory effect of ethylene on rhizobium LCO-induced cell divisions not only plays a role in radial position of root nodules, but also contributes to the lateral inhibitory effect when root nodule formation is initiated.

Root nodule primordium formation is causally linked with the formation of a local auxin maximum [16–20]. This implies that signalling induced by rhizobium interferes with the plant auxin homeostasis. Quantitative modelling studies indicated that a local auxin maximum can be generated upon a local decrease in polar auxin transport [21]. The importance of such a decrease in auxin transport upon rhizobium LCO signalling was shown by two type of experiments. First, it was found that local application of the auxin transport inhibitor TIBA to alfalfa (*Medicago sativa*) roots induces formation of nodule-like structures [22]. Similar results have been achieved in Medicago, using either TIBA or NPA, another auxin transport inhibitor [23]. Second, auxin quantification studies in Medicago wild type as well as in *Mtein2/Mtskl* mutant plants revealed a decrease in auxin transport upon inoculation with rhizobium [24, 25]. Interestingly, the *Mtein2/Mtskl* mutant showed a faster recovery in auxin transport when compared to wild type, and such recovery was associated with an increased expression of the auxin efflux carrier *MtPIN2* [24].

Fate mapping studies in Medicago revealed that the first cell divisions occur in the innermost cortical cell layer and pericycle in a time frame of about 18–24 hours post inoculation [26, 27]. These cells are not in direct contact with the microsymbiont. It is also unlikely that these cells perceive the rhizobial LCOs, as these molecules are very immobile [28]. Furthermore, it has been found that activation LCO receptors whos expression was confined to the epidermis only is sufficient to trigger mitotic activation of cortical cells in both Lotus and Medicago [29, 30]. Taken together, these studies imply that a secondary signal is generated at the root epidermis upon rhizobium LCO signalling that triggers cells in a different location to divide. It remains elusive, however, by which mechanism such secondary signal can trigger a robust local auxin maximum in a way that explains the various nodulation phenotypes of different legumes.

Here we investigate using mathematical modelling how both processes can be linked through a diffusive secondary signal that has an inhibitory effect on auxin efflux. Our model shows that a diffusive signal (DS) produced locally in one epidermal cell is sufficient to trigger the formation of a local auxin maximum in the root inner cell layers. We find that the reduction of auxin efflux by DS leads to an auxin accumulation that occurs first and strongest in the pericycle and the innermost cortical cell layer, both trumping the endodermis that lays between them. It also induces a transient depression of the auxin concentration in the vascular tissue rootward of the DS induction site. This finding is in line with experimental observations and may constitute a lateral inhibitory mechanism to control nodule spacing. We further discuss the core requirements of this mechanism and how they affect the robustness to biological variation of this developmental process.

## Results

### Model outline

**Fig. 1:**
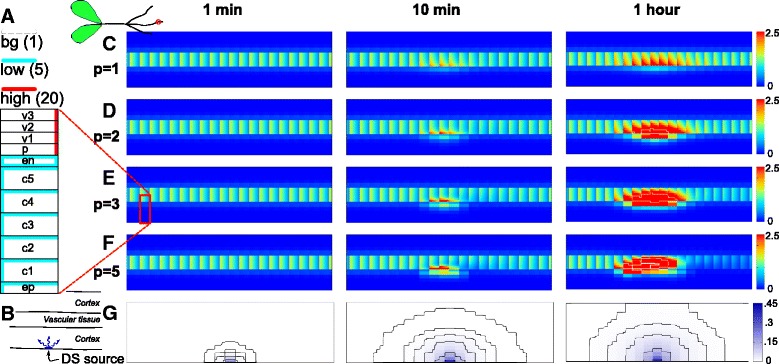
Dynamics of DS induced auxin accumulation. **a** Layout of auxin efflux carriers (PIN proteins) as used in the simulations, shown on 1/2 of the root. Starting levels of PIN (effective efflux permeability *P*_*eff*_) of individual cell faces are one of three: high (red; 20 *μ*m/s), low (cyan; 5 *μ*m/s) or background (bg; no colour; 1 *μ*m/s), following [21]. These layouts result in a rootward auxin flux in the vascular tissue (to the right in these figures) and a less strong shootward counterflux in the cortex. The cartoon plant on top illustrates the orientation of the root segments. **b** Simulation setup: from the start of the simulation, DS is produced continuously in a single epidermal cell and diffuses outward through the root segment. **c–f** Auxin accumulation in response to DS signal produced in a single epidermal cell after 1, 10 and 60 minutes from the start of the simulation. White contours occur at 5 *C*_*v*_ and 25 *C*_*v*_ (bold). *C*_*v*_ is the average vascular auxin concentration before induction. **g** Corresponding concentration profiles of the DS. This is the same for all simulations, regardless of response parameters. Contour lines are plotted at 0.3, 0.1, 0.03, 0.01, 0.003 and 0.0001 a.u. (DS concentration in arbitrary concentration units). All simulations have an overall sensitivity of *h*=100/a.u.. The response steepness is varied from *p* = 1 (**c**), 2 (**d**), 3 (**e**) to 5 (**f**). Movies of C-F are available as Additional files 1, 2, 3 and 4

To investigate how epidermally produced DS can induce a local auxin maximum in the inner root layers, we departed from a previous modelling study [21]. That study addressed the auxin accumulation signatures of different manipulations of auxin transport and metabolism, but left open the question of how these changes could be induced. Here, we modeled the reduction of auxin transport through DS, and investigated the effects of the interaction in the tissue context of the previously developed models of auxin transport in the susceptible zone of legume roots (as recapitulated in Fig. 1 a).

Auxin transport in these simplified root segments is modeled using effective permeabilities for influx (*P*_*inf*_) and efflux (*P*_*eff*_); see methods for details. We used the same value of *P*_*inf*_=20*μ*m/s for all cells and cell faces. Efflux, on the other hand, varied per cell face based on the so-called “PIN layout” of a previously developed model [21]. Following [27], the five cortical cell layers of the model are indicated with C1-C5 starting from the periphery (Fig. 1 a).

We focused on the very first stages of primordium initiation, i.e., before the start of cell divisions. From the start of the simulations, DS was produced at a constant rate in a single epidermal cell. This represents a cell engaging with rhizobium. DS moved diffusively through the cytoplasm and cell walls, and membranes were permeable to it. We used a sigmoidal decreasing function to link DS to reduction of auxin efflux:

1 $$ P_{ef{}f} = P_{ef{}f,intr}/\left(1 + \left(h [DS]\right)^{p}\right).  $$

**Fig. 2:**
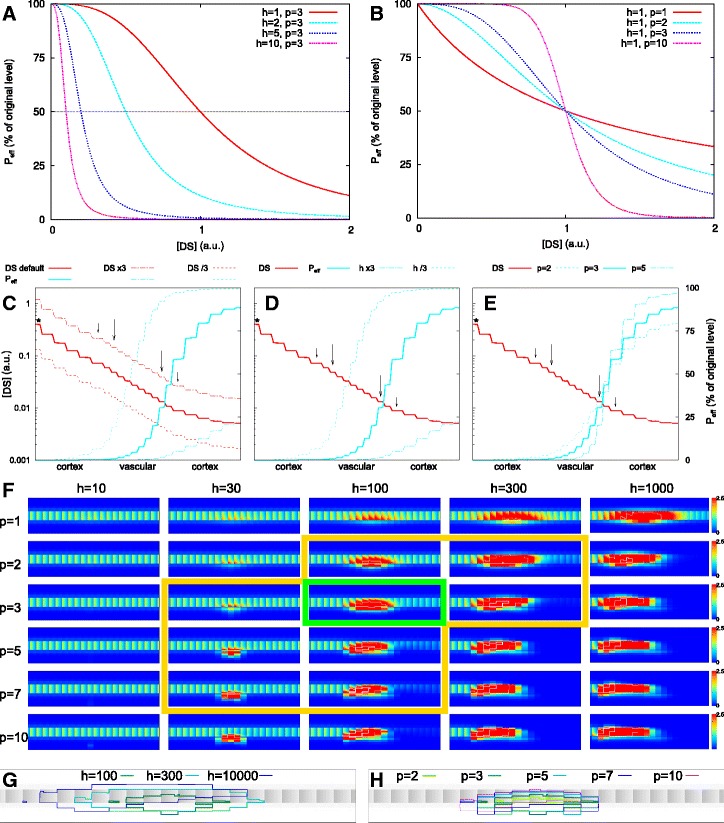
Systematic exploration of response parameters *h* and *p*. **a**, **b** Effects of changing *h* (**a**) and *p* **b** on the response (reduction of *P*_*eff*_). Overall sensitivity *h* moves the location where *P*_*eff*_ is reduced by 50 %, which always occurs at a concentration of 1/*h* (**a**). Response steepness *p* does not affect this point, but changes the steepness of the response (**b**). **c–e** *P*_*eff*_ (in cyan) as percentage of the intrinsic (starting) value as a readout of the axial DS profile through the signaling epidermal cell (*). The steady state DS profile is indicated in red, on a logarithmic scale. Short and long arrows indicate the C5 and pericycle layers, respectively, on both sides of the vascular tissue. **c** Changing DS production by a factor 3 up/down. Line types match DS profile and *P*_*eff*_ response. **d** Changing *h* by a factor 3 up/down. Note that this has the same effect on the readout as changing the amount of DS by the same factor. **e** Changing *p*. Defaults: *h*=100/a.u., *p*=3. **f** Snapshots of auxin concentrations at T = 1 hour for a wide range of *p* and *h* values. Default parameter values are indicated with a green border and reasonable values with a yellow border. For comparability, auxin concentration range for all figures is 0 - 2.5 *C*_*v*_. Where relevant, the white contours occur at 5 *C*_*v*_ (regular) and 25 *C*_*v*_ (*bold*). For full ranges, see Additional file 9: Figure S3. Note that not all segments have reached steady state, although the DS gradients have. **g**, **h** Contour plots of [IAA] = 5 *C*_*v*_ auxin concentration boundary at T = 30 hours with p=3 (**g**), or h=100/a.u. (**h**) fixed. Note that increasing *h* (with constant *p*) can move the boundary arbitrarily far away from the DS producing epidermal cell in the middle of the root segment (**g**), whereas with increasing *p* (with constant *h*) the far end of the boundary saturates (**h**). For additional contours, see Additional file 9: Figure S4

In this, *P*_*eff*,*intr*_ is the starting or “intrinsic” value of *P*_*eff*_ taken from the root layout (Fig. 1 a). *h* is the overall sensitivity of the response: if the DS concentration is [DS] = 1/*h*, the auxin efflux is reduced by 50 %. DS concentration is given in arbitrary units (a.u.) and auxin concentration is given in units relative to the average vascular concentration before induction (*C*_*v*_) [21]. Parameter *p* determines the response steepness: for low values of *p*, an increase of [DS] results in a gradual reduction of the auxin efflux capacity and with increasing values of *p*, the response becomes more step-like (Fig. 2 a, b). This models the interaction between DS and auxin efflux (*P*_*eff*_) in a phenomenological way. We therefore extensively analyzed the robustness of the results with respect to changes in these parameters and the requirements this poses on any biological mechanism operating through similar concepts.

### DS induces a stronger auxin response in pericycle and innermost cortical cell layer than in endodermis

We first explored where and when the epidermal signal would induce local auxin accumulation with various parameters for the DS response. In vivo experiments have shown that LCO signalling can trigger responses in inner cell layers within 1–3 hours post rhizobium LCO-induced signalling, whereas first cell divisions are reported 18–24 hours post inoculation [26, 27, 31, 32]. We, therefore, tested our model for auxin accumulation within a time course of 1 hour from the start of induction (Fig. 1 and Additional files 1, 2, 3 and 4). For all values of response steepness parameter *p*, auxin accumulation occurred in the vascular tissue, and this accumulation became stronger with increasing *p*. Over this 1 hour time interval, strong auxin accumulation only occurred in the inner cortical layers (C5 and to a lesser extent C4), the endodermis and vascular tissue. Among neighbouring cells, the auxin concentration was lower in the endodermis than in both the pericycle, which has direct access to the vascular auxin source, and cortical layer C5, where the DS signal was stronger (Fig. 1 c–e). When we increased simulation time to 18 hours, large increases in the auxin concentration (>1*C*_*v*_, the normal vascular concentration) occurred in additional cortical cell layers, particularly C4 and to a lesser extent C3 (Additional file 9: Figure S1 and movies Additional files 5 and 6). Moving from C5 to more exterior cortical layers, however, the number of cells that reached a particular concentration decreased. These findings are in line with the experimental observation of cell divisions in the Medicago nodule primordium. Xiao et al. [27] found that divisions first occur in the pericycle and cortical layers C5 and C4, and with some delay also in the endodermis and C3. We chose the combination of *h*=100/a.u. and *p*=3 as a default for further investigation based on the resemblance with this developmental data.


Additional file 1: **Supplementary movie 1.** 1 hour time lapse movie of Fig. 2 c. (AVI 765 kb)



Additional file 2: **Supplementary movie 2.** 1 hour time lapse movie of Fig. 2 d. (AVI 831 kb)



Additional file 3: **Supplementary movie 3.** 1 hour time lapse movie of Fig. 2 e. (AVI 860 kb)



Additional file 4: **Supplementary movie 4.** 1 hour time lapse movie of Fig. 2 f. (AVI 852 kb)



Additional file 5: **Supplementary movie 5.** 30 hour time lapse movie of Fig. 2 d/Additional file 9: Figure S1. (AVI 380 kb)



Additional file 6: **Supplementary movie 6.** 30 hour time lapse movie of Fig. 2 e/Additional file 9: Figure S1. (AVI 496 kb)


### Robustness of local auxin accumulation

To test the robustness of the model, we considered two aspects: sensitivity to changes in the model parameters and sensitivity to fluctuations in the DS gradient.

An important evaluation criterion for the model is that auxin accumulation occurs fast enough, that is, well ahead of the experimentally observed onset of cell divisions. Without local auxin production, it could be possible that the rate of local auxin accumulation is severely limited by the rate of auxin supply from other parts of the plant. To assess the importance of the auxin supply rate for the timely accumulation of auxin, we performed simulations with decreased or “slowed down” auxin transport dynamics [21]. For this, all influx and baseline efflux effective permeabilities were decreased tenfold (Additional file 9: Figure S2 and movies Additional files 7 and 8).


Additional file 7: **Supplementary movie 7.** 30 hour time lapse movie of Additional file 9: Figure S2A-F. (AVI 598 kb)



Additional file 8: **Supplementary movie 8.** 30 hour time lapse movie of Additional file 9: Figure S2G-I. (AVI 143 kb)


Most importantly, with this decreased auxin transport activity, we still observed a strong auxin accumulation in the pericycle and inner cortex within 1 hour. As a second order effect, we observed that the maximum auxin concentrations reached were somewhat higher. Correspondingly, the area that could reach a given concentration was slightly larger. Consistently, the timescale for approaching a steady state auxin maximum increased similarly more than tenfold, in line with the larger total auxin accumulation.

We then investigated how the response to DS affects the characteristics of auxin accumulation. In our model, the driver of auxin accumulation is the reduction of auxin transport by DS, so we plotted the reduction of *P*_*eff*_ levels, for different *h* and *p* against a linear gradient of DS (Fig. 2 a, b) and the axial DS profile measured from our previous simulations (Fig. 2 c–e). Comparing Fig. 2 c and d shows that a change in DS production rate had the same effect as the corresponding change in overall sensitivity *h*. This is because in our model, a change in the production rate changes the amount of DS by the same factor everywhere. Consequently, one can maintain identical auxin patterns by simultaneously increasing DS production by a factor *x* and decreasing the overall sensitivity *h* to 1/*x* times its original value. The biological implication is that the amount and spreading of the signal should match the sensitivity of the response to it.

Next, we performed multiple simulations varying both *h* and *p*. Increasing *h* and *p* both resulted in a broadened zone of auxin accumulation (Fig. 2 f, Additional file 9: Figure S3), however, with a clear difference. Increasing *h* steadily moved the boundary of the auxin maximum farther away from the inducing epidermal cell (Fig. 2 g, Additional file 9: Figure S4A-D). In contrast, with increasing *p*, the expansion of the maximum saturated (Fig. 2 h, Additional file 9: Figure S4E-G). This difference can be understood from the basic response curves (Fig. 2 a, b). Changing *p* makes the response curve steeper (Fig. 2 b, e), but does not affect the 50 % reduction point (1D) or boundary (root model). Therefore, increasing *p* can only increase primordium size up to the location of this boundary. The boundary resides at the position where [DS] = $\frac {1}{h}$ (Fig. 2 a), i.e., it is determined by the DS gradient and *h* together, so changing *h* moves this boundary. The larger the overall sensitivity *h*, the farther away from the source the DS signal can be detected.

With the DS gradient parameters we used, *h* had to be at least ≈ 30/a.u. for a response in the pericycle. From such values onward, increasing *h* resulted in more C5 cortical cells that received a sufficiently large DS concentration to accumulate auxin. The time needed to reach a high auxin concentration in all these cells, however, became very long for high values of *h* and *p* (≫30 hours; Additional file 9: Figure S4). At the same time, the zone with the highest auxin concentration on relevant time scales moved shootward relative to the site of DS production. This implies that a too sensitive response to DS will result in a spatial mismatch between the sites of primordium induction and rhizobium entry (right column of Fig. 2 f and Additional file 9: Figure S3; Fig. 2 g, Additional file 9: Figure S4).

**Fig. 3:**
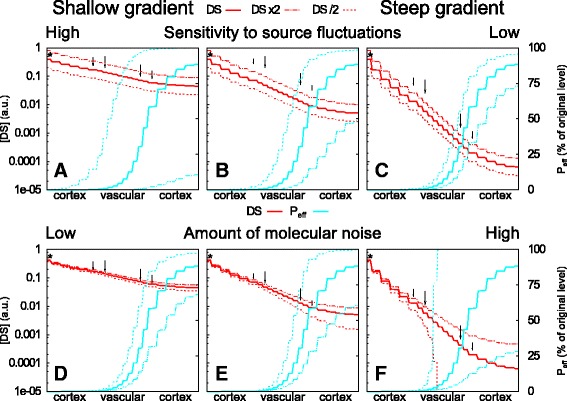
Robustness of mechanism with regard to changes in the overall amount of DS (**a–c**) and molecular noise (**d–f**). The DS profile on a axial line through the producing cell (*) is indicated in red, the corresponding “readout”, *P*_*eff*_ as a percentage of the intrinsic value, is indicated in cyan. The C5 and pericycle cells are indicated with short and long arrows, respectively. **a–c** Change of production rate by a factor 2 from the original moves the original profile (solid line) up or down by the same factor everywhere (*dashed lines*). **b** Default steady state DS profile. **a** Half as steep profile. **c** Twice as steep profile. **d–f**: Impact of molecular noise dependent on gradient steepness. Noise is illustrated by the lines $[DS] \pm 0.05 \sqrt {[DS]}$. The overall sensitivity (*h*) and response steepness *p* are adjusted to keep the primary response curve identical: *h*=15.797/a.u., *p*=6 (**a**, **d**), *h*=100/a.u., *p*=3 (**b**, **e**; default) and *h*=4007.2/a.u., *p*=1.5 (**c**, **f**)

A very important feature of a functional primordium induction mechanism is that it is robust to normal biological fluctuations in the amount of signal. We, therefore, investigated how fluctuations in the DS gradient affect the reliability of primordium positioning. Figure 3 a–c shows how a factor two increase or decrease in the production rate of DS, which shifts the whole steady state gradient up or down, respectively, translated to larger or smaller shifts in the “responsive region” depending on the steepness of the gradient. Steeper gradients resulted in more robust positioning of the responsive region with regard to changes in the total amount of DS.

A steeper gradient, however, has a larger relative concentration difference between source and the 50 % response point. In other words: given the concentration at the source, the number of DS molecules that reaches the inner cell layers decreases with increasing gradient steepness. The impact of molecular fluctuations depends on *N*, the number of molecules involved. As molecular fluctuations scale with $\sqrt {N}$, the signal-to-noise ratio also scales with $\sqrt {N}$, and *N* is proportional to the local DS concentration. This effect is illustrated in Fig. 3 d–f. Because the DS concentration is in arbitrary units, the number of molecules is unspecified. We therefore arbitrarily fixed the molecular noise as 0.05 * $\sqrt {[\text {DS}]}$ for all three figures.

The combination of both types of robustness suggests that there is some optimal gradient steepness. This optimum balances a trade-off between robustness against on the one hand changes in e.g. production rate and parameters that affect the spread of the signal, and on the other hand the amount of noise in the detection of the signal.

In plants, the DS gradient could be modified in several ways. First, by changing the membrane permeability to DS. The chemical nature of DS dictates a minimum permeability, which might be enhanced by specific transporters. Second, through the turnover or sequestration of DS in the cytoplasm of intervening cells. Higher turnover produces steeper gradients. Third, by variation of the number of cell layers between the DS source and the target cells. Model legumes such as Medicago and Lotus have a stereotypical number of cortical layers, so variation of this kind is negligible. In Figs. 2 c–e and 3 we observed stepwise DS gradients, implying that intracellular DS diffusion is not limiting the spread of the signal. (This happens in the model, because intracellular DS diffusion is fast (*D*=200*μ*m^2^/s) relative to crossing to the neighbouring cell (effective permeability of 2 membranes and the wall together ≈0.5*μ*m/s)). Such stepwise gradients suggest that matching DS signal and response in the root inner layers is hard in species with high within species variation of the number of cortical cell layers in the susceptible zone. If such species exist, they would require an additional mechanism for response confinement. To our knowledge, however, such extensive within species variation has not been described yet.

### Induction of determinate nodules

**Fig. 4:**
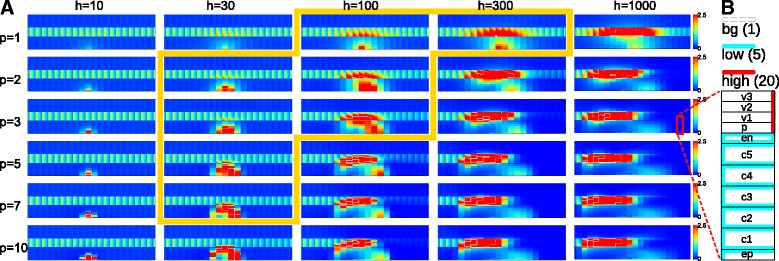
DS induced auxin accumulation in the outer cortical layers in a background with a larger cortical auxin supply. **a** screen over different *h* and *p* values as in Fig. 2 f, but with a different PIN layout (B; see also: [21]) that results in higher cortical auxin availability than with the default PIN layout. Because of this higher cortical auxin availability, the DS signal also induces substantial auxin accumulation in the outer cortical layers. All figures are snapshots at T = 1 hour. The *yellow border* indicates reasonable parameter values for cortical auxin accumulation. **b** PIN layout. In absence of DS, *P*_*eff*,*intr*_, the intrinsic effective efflux permeability, is one of three levels, as indicated: high (*red*) = 20 *μ*m/s, low (*cyan*) = 5 *μ*m/s, bg (*“background”, white*) = 1 *μ*m/s

Different legume species make different nodules. So far, we have focussed on Medicago type indeterminate nodules. Legumes in the Dalbergioid (e.g. lupin), Millettioid (e.g. soybean) and Loteae (e.g. Lotus) clades form so-called determinate nodules that originate from the outer or middle cortical layers, respectively [33, 34]. As in indeterminate nodules, auxin accumulation occurs at the site of cell divisions [17, 20]. In our previous modelling study [21], we have found that auxin accumulation occurred in a more exterior cortical position in case of a different distribution of cortical PINs (Fig. 4 b), which leads to a higher cortical auxin availability. Using the same alternative PIN layout, we now investigated how the cortical PIN distribution affects the axial position of auxin accumulation (Fig. 4, Additional file 9: Figure S5). We observed different effects depending on the sensitivity of the DS response (parameter *h*). We found that DS could induce either only a cortical maximum (*h*≈30/a.u.), or both a cortical and a vascular maximum when the response to DS was more sensitive (*h*≈100/a.u. or larger). Generally, the vascular auxin concentration during primordium induction behaved very similar to the default “indeterminate” case. This is in line with expectations, because the vascular tissue itself is the main auxin source in our simulations.

### Transient reduction of rootward vascular auxin concentration contributes to nodule number control

**Fig. 5:**
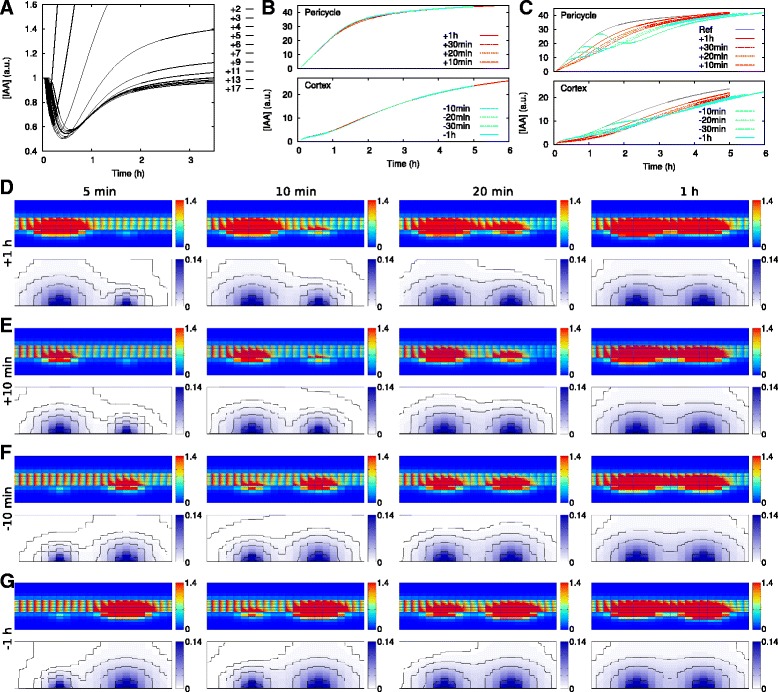
Competition for auxin among nearby primordia. **a** Quantification of the reduction of the auxin concentration in the pericycle downstream of the induction site (labels indicate the distance in number of cells; the position where the second (downstream) primordium would be induced is indicated with a thick line (“+9”)). **b**, **c**: Auxin concentration in (the center of) the pericycle cell and C5 inner cortical cell closest to the DS production site upstream (**b**) or downstream (**c**) at time since the initiation of the respective primordium. Line labels are shared between top and bottom panels: *red* lines indicate the upstream primordium was initiated first and *cyan* lines indicate the downsteam primordium was initiated first. The concentration increase in absence of a second primordium is indicated by *dotted-dashed gray* lines in (**c**). **d–g**: Labels on the *left* indicate how long before the downstream (*right*) primordium the induction of the upstream (*left*) primordium was initiated, labels on the *top* indicate time since the initiation of the second primordium. **d**, **e** Upstream primordium induced first. **f**, **g** Downstream primordium induced first

In the time lapse movies of the simulations (Additional files 1, 2, 3, 4, 5, 6 and 7), we observed a temporal decrease of the auxin concentration rootward of the induced primordium, which was more pronounced and lasted longer with increasing values of *h* and/or *p*. To estimate its importance, we quantified the extent and duration of the depression by following the auxin concentration over time in the pericycle cells downstream of the epidermal induction point (Fig. 5 a, Additional file 9: Figure S6). With default parameters (*h*=100/a.u., *p*=3), downstream cells would need up to 2 hours to recover to almost the starting level and more to fully recover. Over that period, the primordium acted at as an auxin sink, tapping on the rootward flux through the vascular tissue, and reducing the amount of auxin available downstream.

As the vascular tissue is the main auxin or only supply for early stage primordia, the developmental auxin depression caused by an early stage primordium could delay the formation of additional primordia downstream (i.e., rootward) of it. To investigate this inhibiting effect, we induced a second primordium by DS production in a second epidermal cell, nine cells upstream or downstream of the first producing cell, starting 10, 20, 30 or 60 minutes later (Fig. 5 b–g). Quantifying the auxin concentration in the inner cortex (C5) and pericycle, in the cells closest to the respective induction points, we found that the upstream (shootward) primordium was barely affected by the other primordium (Fig. 5 b), whereas in the downstream primordium auxin accumulation was delayed (Fig. 5 c). We even observed a temporal concentration decrease in the pericycle when the upstream primordium was induced 30 or 60 minutes after the downstream primordium (Fig. 5 c).

**Fig. 6:**
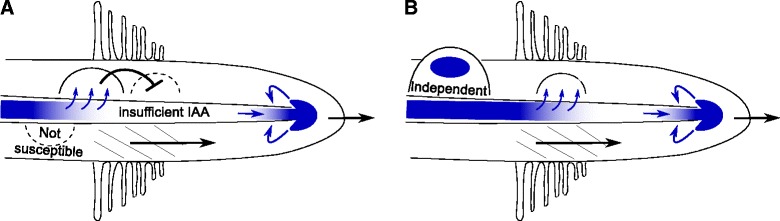
Outline of partial nodule number control mechanism. Our observations of the transient rootward auxin depression during primordium initiation suggest the following mechanism that contributes to the spatial separation of nodules. **a** An early stage primodrium serves as an auxin sink (Figs. 5, Additional file 9: Figure S6), which temporarily reduces the auxin availability downstream (*rootward*) of it. This temporarily inhibits the formation of new primordia. The forming primordium will not be deprived of its auxin source by the initiation of a new upstream (*shootward*) primodrium, because there, the root is no longer susceptible: the susceptible zone (hatched area) moves rootward as the root continues its growth. **b** As the primordium matures, it stops functioning as a net auxin sink (i.e., becomes “independent”) and auxin supply is restored to the susceptible zone (at its progressed location). Local auxin reflux ensures auxin-autonomy at the root tip (c.f. [66])

This inhibition of auxin accumulation could form part of a mechanism for nodule discretization and limiting nodule density: the susceptible zone forms a window that slides rootward as the root continues to grow and develop, which limits the induction of shootward (upstream) primordia. At the same time, currently developing primordia can temporarily reduce the downstream susceptibility by limiting auxin supply, rendering the modelled mechanism for first induction of auxin accumulation less effective (Fig. 6).

## Discussion

We have modelled the first stages of the formation of a legume indeterminate root nodule triggered by a diffusive signal of epidermal origin. This signal induces a local auxin maximum by inhibiting auxin efflux activity. Although we have made the worst-case assumption that all cells respond in the same way to this hypothetical diffusive signal, we found that the induced reduction of auxin transport caused the largest auxin accumulation in the inner root layers, particularly the pericycle and innermost cortical layer. These layers accumulated most auxin, because of a favourable combination of the strength of the diffusive signal that reached them, and their position relative to the auxin sources that are present in the root.

The observed patterns of auxin accumulation in our model explain a number of observations in cell fate mapping of nodule organogenesis in Medicago [27]. There, divisions in the endodermis were delayed relative to the flanking pericycle and innermost cortical cell layer. Correspondingly, we consistently observed stronger auxin accumulation in both pericycle and the C5 cortical layer than in the endodermis. Moreover, it was reported that the onset of cell divisions in the third cortical layer (C3) occurred later than in more inner cortical cell layers (C4/C5). Assuming that cell divisions occur a given time after reaching a certain threshold in auxin concentration, our model also predicts such a temporal difference in onset.

### Mechanisms for nodule spacing

We observed a temporal decrease of the vascular auxin concentration rootward of the diffusive signal production site. In our model, this temporal reduction of auxin availability suppressed the induction of subsequent downstream primordia by reducing auxin availability (Fig. 5). We argue that this mechanism contributes to the spacing (and density) of root nodules (Fig. 6). The mechanism is complementary to and directly compatible with the phenotypes observed in the Medicago, Lotus and soybean (*Glycine max*) hypernodulation mutants *Mtsunn, Ljhar1,* and *Gmnark* [7, 35, 36]. These mutants are defective in the autoregulation of nodule number and produce large numbers of small root nodules over the whole root system. *MtSUNN, LjHAR1,* and *GmNARK* belong to the same orthology group as *A. thaliana CLAVATA1* (*AtCLV1*), and encode a Leu-rich repeat receptor-like kinase [35, 36]. In legumes, these proteins are involved in the reduction of auxin loading in the shoot following successful nodulation events [37]. Both mechanisms, the rootward auxin depression during primordium initiation and MtSUNN/LjHAR1/GmNARK action, result in decreased auxin availability in the root susceptible zone, albeit likely at different time scales. MtSUNN/LjHAR1/GmNARK activity in the shoot is induced by mobile CLE peptides that are produced in the root upon inoculation [38]. Production and transport of these signals result in a delayed effect on root auxin content. The rootward auxin depression during primordium initiation, on the other hand, occurs in our model within 30 minutes with default parameter settings (Fig. 5 a) and for all values of *p* and *h* tested (Additional file 9: Figure S6). For each parameter combination, the rootward auxin depression reduces the downstream nodulation potential for a ±fixed time. Towards higher response steepness (*p*) and overall sensitivity (*h*), or if more diffusive signal is produced, the predicted rootward auxin depression lasts longer. During this long period, several events may have occurred in the root that we do not consider in our model. Among these, the cortical cell divisions that will give rise to the nodule primordium are most prominent. We argue that the local auxin maximum of such developing primordia becomes self sustaining and independent of the vascular auxin source within this time frame, resulting in faster recovery of the downstream nodulation potential.

Besides a rootward depletion of auxin triggered by the formation of a nodule primordium, additional negative feedback loops are activated. For example, ethylene –for which the biosynthetic pathway is activated upon LCO signalling [39]– inhibits the nodulation response effectively [40]. The ethylene insensitive Medicago mutant *Mtein2/Mtskl* shows clusters with a high density of small nodules or even merged primordia [6, 8, 14]. These observations suggest that local inhibition of nodulation by a single (or a few) nodules is incomplete in the mutant. *Mtein2/Mtskl* mutant plants also have increased root auxin content and transport [24], which could further explain the nodule clustering phenotype. The clusters are nevertheless separated by zones without nodules, indicating periods of auxin shortage. This shortage could be caused by the collective sink strength of a large number of small nodules developing simultaneously, or by shoot (MtSUNN/LjHAR1/GmNARK) regulated auxin reduction.

The negative feedback from ethylene (and other signals) could make the induction of nodule primordia more robust against variations in the amount of DS produced and in the strength of the response to the signal. Nevertheless, even the Medicago *Mtsunn, Mtein2/Mtskl* double mutant, which is defective in both known mechanisms for the control of nodule number, can produce discrete nodules. This may imply that vascular auxin depletion during primordium initiation indeed contributes to the discretization of nodule primordia.

### Indeterminate versus determinate nodules

Contrary to indeterminate nodules, determinate nodule primordia are formed from the middle to outer cortical layers [34] corresponding with auxin accumulation in exterior root layers [17]. In biological terms, our simulations suggest two scenarios for the initiation of determinate nodule primordia. First, it is possible that determinate and indeterminate nodule primordia are formed with the exact same response to the diffusive signal (same parameters), and that the cortical auxin availability determines the axial position of the primordium (c.f. [21]). Under this scenario, our simulations predict that a depression of the vascular auxin concentration, as is experimentally observed in Medicago, also occurs in legumes forming determinate nodules. In the alternative scenario, determinate nodule primordia are induced by a weaker response to the diffusive signal (≈lower *h*) in combination with a higher auxin availability in the outer cortical layers. Under the latter scenario, little or no auxin accumulation will occur in the vascular tissue. This implies that the spacing mechanism that we suggested based on our simulations of indeterminate nodule primordium formation would not work in case of determinate nodules. Both scenarios for determinate nodule primordium formation are, in principle, experimentally distinguishable by monitoring the vascular auxin concentration post inoculation. Experiments in Lotus using auxin responsive GH3 promoter reporter constructs indicated increased GUS and GFP activity in the vasculature at the sites of formation of (determinate) nodules [17]. This finding supports the first scenario, in which the axial position of the primordium is determined by the cortical auxin availability (c.f. [21]). Moreover, at 2 days post inoculation, GH3 activity has been observed on both the apical and basal side of the Lotus nodule primordium [20].

### Concentration dependent LCOs responsiveness

Work with exogenous LCO application shows that the induction of cortical cell divisions requires higher concentrations of LCOs than epidermal responses (such as root hair deformations and symbiotic gene expression) [41]. Our model can explain this difference in response if we make two assumptions. First, that the epidermal responses are independent of the diffusive signal, second, that the amount of the diffusive signal that is produced in the epidermis depends on the amount (and molecular signature) of the LCOs detected. A low amount of LCO would then result in little or no diffusive signal. In our model, lowering the DS production is equivalent to lowering the overall response sensitivity parameter *h*, for example to *h*=10/a.u. or less. As can be seen from the left side of Figs. 2 f and 4, such low amounts of DS would be insufficient to trigger auxin accumulation in the inner cortex and pericycle. Moreover, with the default PIN layout, there is so little auxin available in the outer cortex, that we observed no detectable auxin response at all (Fig. 2 f). This two-step response to LCOs is supported by genetic dissection studies in Medicago and Lotus. For example, the cytokinin receptor mutants *Mtcre1* and *Ljlhk1* are blocked in primordium formation, whereas epidermal responses can occur normally [42, 43]. Additionally, cortical overexpression of transcriptial regulator NODULE INCEPTION (NIN) [44] in Medicago can induce spontaneous nodule-like structures, even in *Mtnin* and *Mtcre1* single mutants [32], which demonstrates that epidermal and cortical responses are distinct processes.

### Candidate molecules for DS

The model presented here does not specify the chemical nature of DS. Several signaling molecules have been implicated in nodule formation that may act as a diffusive signal; e.g. calcium (Ca ^2+^), strigolactone, cytokinin and flavonoids [45–48]. Early experiments using fluorescent dyes have demonstrated that Ca ^2+^ signals can travel through plant tissues as waves through excitable media [49]. Experiments measuring the frequency of Ca ^2+^ spiking in the epidermis and outer cortex of Medicago during the formation of infection threads, however, show different temporal profiles in neighbouring cells [50]. This suggests that spiking frequencies are not readily communicated to neighbouring cells or more interior cell layers, but rather are interpreted as local cellular symbiotic responses. This makes it unlikely that Ca ^2+^ fulfills a function as diffusive signal in a symbiotic context. The mobility of cytokinins, strigolactones and/or flavonoids is more suited to fulfill such function. Strigolactones are known to interfere with PIN protein levels [51–53]. Application of the synthetic strigolactone analog GR24 results in an increased number of nodules in *Medicago sativa* [54]. Several mutants in strigolactone biosynthesis and response are available in *Pisum sativum*, a legume that produces indeterminate nodules. Of these, the *Psrms1* mutant (defective in the homolog of *Arabidopsis thaliana* (Arabidopsis) *AtMAX4*) contains almost no strigolactones, but produces only 40 % fewer nodules than wild type [46]. Similar results are found in Lotus, which produces determinate nodules: silencing of *LjCCD7* (homologous to Arabidopsis *AtMAX3*) by over 70 % resulted in a greatly increased root mass and a mere 20 % reduction of nodule number per gram of root fresh weight [55]. These results suggest that strigolactones may be involved in nodule number control, but are not essential for their formation, making strigolactones an unlikely candidate for DS.

In Medicago roots, the cytokinin concentration rapidly increases upon LCO signalling [39]. Furthermore, local cytokinin application induces nodule-like structures on legume roots [47]. In Arabidopsis, exogenous cytokinin is associated with reduction of PIN proteins from the cell membrane resulting in a reduction of auxin efflux [56–59]. Medicago roots of the cytokinin insensitive receptor mutant *Mtcre1* contain larger amounts of polar PIN proteins in the cell membrane than wild type. They, moreover, do not show the decrease in polar auxin transport after inoculation with rhizobium observed in wild type [43]. This indicates that the effect of cytokinin on PIN proteins requires an active cytokinin signalling machinery. Likewise, nodule formation depends on this receptor, which is active in the root inner cells layers [42, 43, 60, 61]. Moreover, a gain-of-function mutation that renders this receptor hypersensitive to cytokinin leads to spontaneous formation of pseudonodules [12, 13]. Moreover, in a Medicago study with the cytokinin responsive *TCSn:GUS* reporter construct, GUS activity was strongest in the epidermis and decreased towards the root’s center at 8 hours post inoculation [62], in line with the decreasing DS gradient in our model. Additionally, application of specific flavonoids can rescue nodule formation in the Medicago mutant *Mtcre1* [25]. This suggests that flavonoid signaling acts downstream or independent of cytokinin signaling. It also shows that flavonoids can reach the inner root layers from the epidermis. It is unclear, however, whether flavonoids would also bypass cytokinin signaling under normal conditions. Taken together, this makes cytokinin and/or flavonoids good candidates to function as diffusive signal. The two signals may act in conjunction.

## Conclusion

To study the induction of nodule primordia, we have modelled the interaction between a diffusive secondary signal and auxin using a phenomenological model of induced reduction of auxin efflux (PIN proteins) in response to DS. The modelled mechanism is able to induce auxin accumulation primarily in the inner cortex and pericycle in response to a signal of epidermal origin, even if the intervening cells all respond to the signal in the same way. Two experimentally observed phenomena emerge from the model: 1) a response that is stronger in the inner cortex and pericycle than in the endodermis, consistent with a recently published fate map of early cell divisions in *M. truncatula*; and 2) a transient depression of the auxin availability rootward of the primordium.

## Methods

### Setup

In our simulations, DS was produced in a single epidermal cell in the middle of the simulation domain, or in two cells equally far from the middle in Fig. 5. Root segments representing the susceptible zone were created based on an Arabidopsis model [63] and adjusted for the Medicago geometry by adding extra cortical layers as previously described [21], but twice as long (48 cell lengths). All cells were 100 *μ*m long and 20 *μ*m (cortical cells) or 10 *μ*m wide (all other cells).

### Parameters

Default auxin transport and metabolism parameters were the same as in [21]. DS was produced with rate 0.01 a.u./s in the designated cell(s), and degraded with rate 0.001/s in all cells. Auxin serves as an important shoot-to-root long distance signal, so we assumed that auxin degradation within the root segment is negligible. Within cells and inside walls, both auxin and DS move by diffusion. Diffusion constants were 200 or 300 μ*m* ^2^/s within cells and 30 or 44 μ*m* ^2^/s in walls for DS and auxin, respectively. The DS values are 2/3 of the auxin values to take into account that auxin molecules are smaller than likely DS candidate cytokinin. Auxin transport over membranes was modelled using effective permeabilities for influx (constant *P*_*inf*_) and efflux (*P*_*eff*_; This generates an outward flux of $\vec {J} = (P_{ef{}f} C_{in} - P_{in} C_{out})\vec {n}$ per unit of membrane surface area, where *C*_*in*_ and *C*_*out*_ are the intracellular and extracellular concentration at the respective membrane segment, respectively, and $\vec {n}$ is the outward pointing normal vector. The maximum *P*_*eff*_ of each membrane segment was taken from the root PIN layout (Fig. 1 a) and decreasing with increasing DS concentration in the cell (Eq. 1)). By default, *P*_*inf*_=20 μm/s for all cell faces. We assumed that DS transport over the membrane is passive, with a membrane permeability of 1 *μ*m/s for DS. This is almost twice the value typically used for protonated auxin (IAAH) (e.g. see [64]), and takes into account the uncharged nature of cytokinin. In Additional file 9: Figure S2 all (starting) values of *P*_*eff*_ and *P*_*inf*_ are one tenth of the default values.

### Numerical methods

We used the Alternating Direction Implicit (ADI) algorithm [65] with a full time step of 1 second (up to 1 hour) or 2.5 seconds (up to 30 hours). Diffusion, degradation, production and transport were calculated for auxin and DS separately. To minimize the discrepancy between DS concentrations and auxin transport parameters, the latter were updated using the average DS concentration per cell after every half time step of the ADI algorithm. We used a square simulation grid with a pixel size of 2 ×2 *μ*m^2^ inside cells, deformed to accommodate a wall thickness of 0.2 *μ*m.

## Additional files

Additional file 9
**Supplementary figures S1 to S6.** (PDF 1110 kb)

## Abbreviations

a.u.: Arbitrary unit; used for DS concentration
*C*_*v*_: Unit for auxin concentration: the average concentration in the vascular tissue before the start of DS production
DS: Diffusive signal
*h*: Model parameter, overall sensitivity of response to DS
LCOs: Lipo-chitooligosaccharides
*p*: Model parameter, response steepness
*P*_*eff*_: Model parameter, effective efflux permeability for auxin transport, modelling the effect of PIN proteins in the membrane
*P*_*inf*_: Model parameter, effective influx permeability for auxin transport, modelling passive permeability and Aux1/LAX based influx in one process
