# Supplementary material for: Quantitative modelling of legume root nodule primordium induction by a diffusive signal of epidermal origin that inhibits auxin efflux
Source: BMC Plant Biol. 2016 Nov 15;16:254. doi: 10.1186/s12870-016-0935-9 (PMC5109694; doi:10.1186/s12870-016-0935-9)
Supplement: Additional file 9 — Supplementary figures S1 to S6. (PDF 1110 kb) [file 12870_2016_935_MOESM9_ESM.pdf]

RESEARCH

Quantitative modelling of legume root nodule primordium induction by a diffusive signal of epidermal origin that inhibits auxin efflux

Eva E Deinum[1*], Wouter Kohlen[2] and René Geurts[2]

[*]Correspondence: eva.deinum@wur.nl
[1]Mathematical and Statistical methods group, Wageningen University, Droevendaalsesteeg 1, 6708 PB Wageningen, the Netherlands
[2]Laboratory for Molecular Biology, Wageningen University, Droevendaalsesteeg 1, 6708 PB Wageningen, the Netherlands
Full list of author information is available at the end of the article

Abstract

Supplementary information: figures S1 to S6

Additional Files available online

Additional file 1 — Supplementary movie 1
1 hour time lapse movie of fig. 2C

Additional file 2 — Supplementary movie 2
1 hour time lapse movie of fig. 2D

Additional file 3 — Supplementary movie 3
1 hour time lapse movie of fig. 2E

Additional file 4 — Supplementary movie 4
1 hour time lapse movie of fig. 2F

Additional file 5 — Supplementary movie 5
30 hour time lapse movie of fig. 2D / S1

Additional file 6 — Supplementary movie 6
30 hour time lapse movie of fig. 2E / S1

Additional file 7 — Supplementary movie 7
30 hour time lapse movie of fig. S2A-F

Additional file 8 — Supplementary movie 8
30 hour time lapse movie of fig. S2G-I

Additional file 9 — Supplementary figures S1 to S6
(this file)

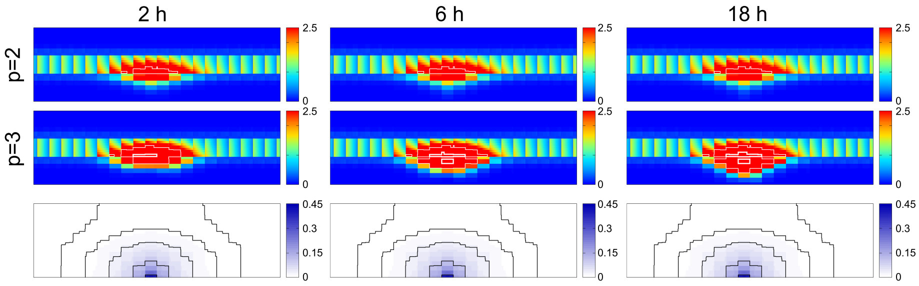

Figure S1 Auxin accumulation at longer time scales With $p = 2$, auxin accumulation has equilibrated within $\approx$ 2 hours, with a distinctly elevated auxin concentration in cortical layers C5, and to a lesser extent also C4. With $p = 3$, the area of large auxin accumulation continues longer and grows further outward. (The 18 hour snapshot should be interpreted with caution, because this is only a short time before the first cell divisions. Changes of cell polarity may have occurred, as well as expression of different proteins involved in auxin transport.) Parameters: $h = 100/a.u.$, $p = 2$ (top) or $p = 3$ (bottom), defaults. White contours 5x and 25x (bold) C_v, the average vascular concentration before induction. See also Additional files 5 and 6.

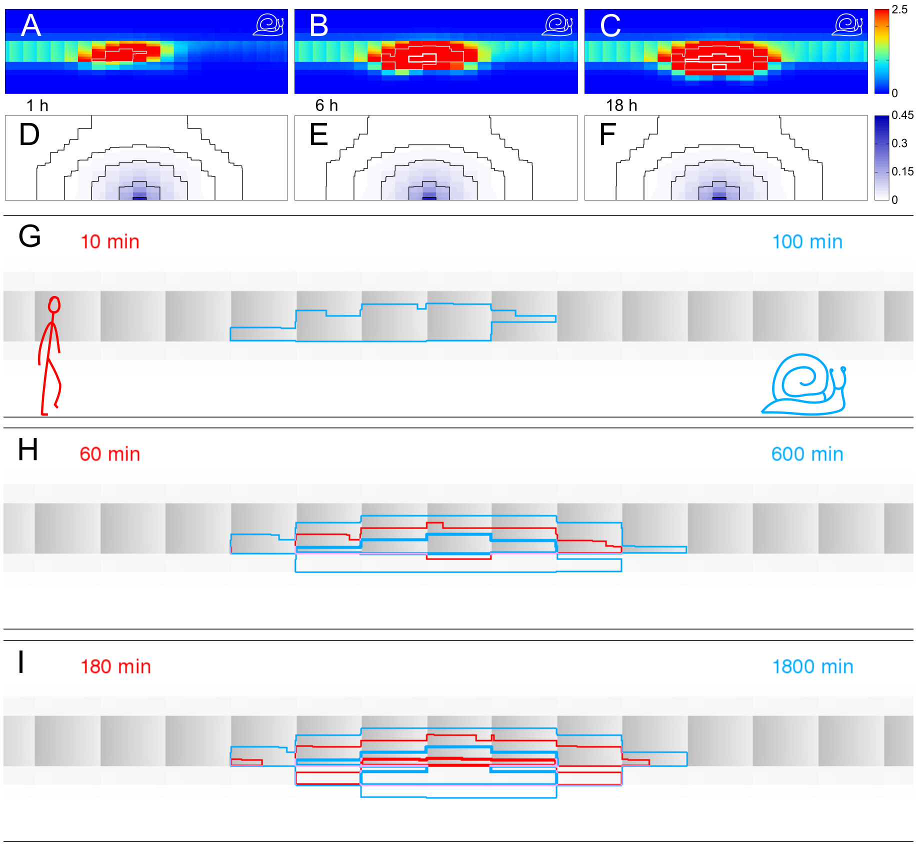

Figure S2 Auxin accumulation with reduced ("slowed down") auxin transport A-C: Induction of auxin accumulation in case of slowed down auxin transport dynamics ($P_{infl}/10$, $P_{effl}/10$: 10x lower effective influx and efflux permeabilities). Auxin accumulation still occurs fast enough to be compatible with observed cytological events. D-F: Corresponding DS concentration profiles. See also supplementary movie 7. G-I: Comparison of default (red) and slowed (blue) auxin dynamics ten times later than default. At matched time points, the contours should be similar. Thin contours indicate the 5x C_v and thick contours indicate the 25x C_v boundary of auxin accumulation. Overlap between contours is drawn in pink. The slowdown of auxin transport resulted in increased auxin concentrations within the primordium and hence wider contours. Otherwise, the shape of the induced auxin maximum at comparable time points and its degree of left-right asymmetry were similar. Auxin concentrations before induction (default parameters) are indicated in gray, to show the position of root tissues. Regular contours indicate 5 C_v, bold contours 25 C_v. See also Additional file 8.

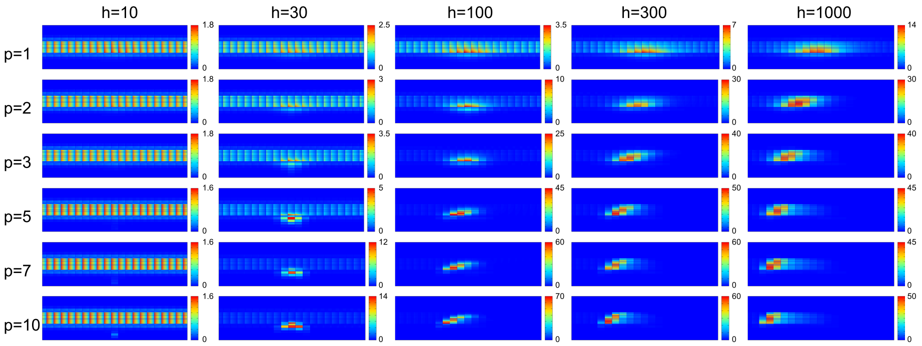

Figure S3 Repetition of figure 2F, but with variable scales for auxin concentration to show the location of largest auxin accumulation. The maximum concentration at 1 hour typically occurs in the vascular tissue and close to the shootward (left) end of the zone responding to the DS signal. Note that for $h = 1000/a.u.$, this is up to 6 cells shootward of the signaling cell, i.e., ≈twice the distance between center and edge of a typical primordium. All figures are snapshots at T $=$ 1 hour.

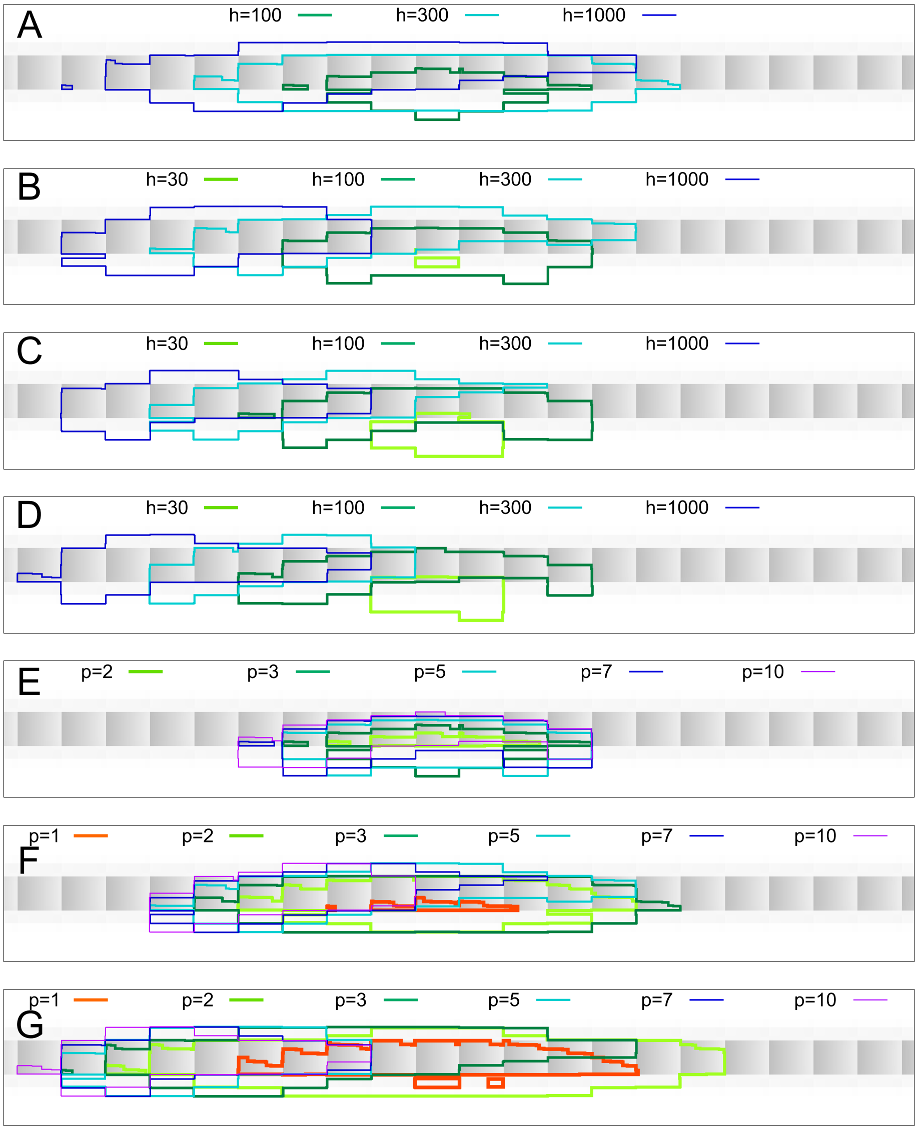

Figure S4 Additional contour plots for comparing the effects of changing p and h on the region where (large) auxin accumulation is induced, similar to figure 2GH. Contour plots of [IAA] $= 5\ C_v$ auxin concentration boundary with $p = 3$ (A), $p = 5$ (B), $p = 7$ (C), $p = 10$ (D), $h = 100$/a.u. (E), $h = 300$/a.u (F) and $h = 1000$/a.u. (G). All contours are taken at T = 30 hours.

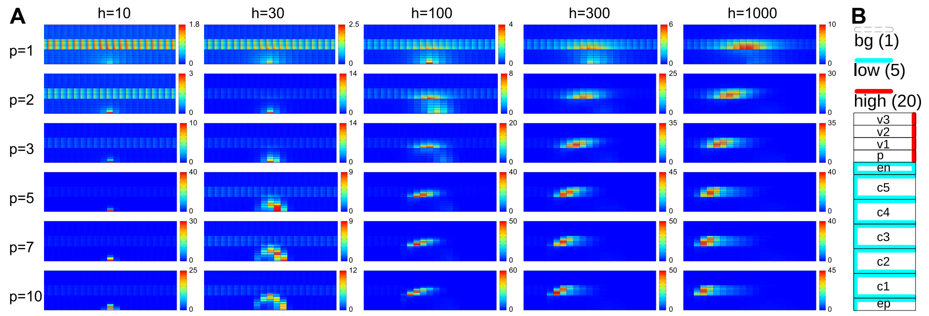

Figure S5 Screen of h and p with PIN layout that causes high cortical auxin content, similar to figure 4, but with varying concentration range. A: Auxin concentration gradients rescaled for each figure to accommodate the full range of concentration variation, as indicated. All figures are snapshots at T = 1 hour. B: PIN layout. In absence of DS, the intrinsic effective efflux permeability ($P_{eff,intr}$) is one of three levels, as indicated. High (red) = 20 $\mu m/s$, low (cyan) = 5 $\mu m/s$, bg ("background", white) = 1 $\mu m/s$.

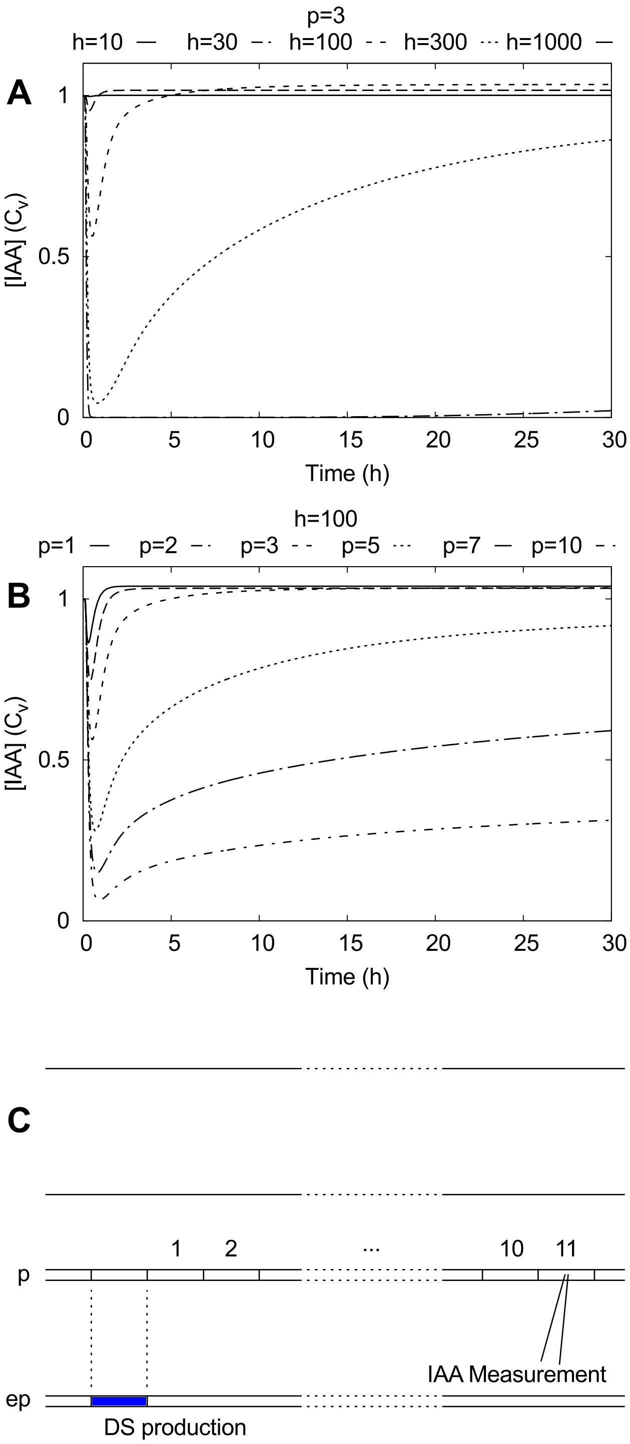

Figure S6 Quantification of rootward auxin depression during primordium initiation. Curves show auxin concentrations over time in the pericycle cell 11 cells rootward of the pericycle cell closest to the signaling epidermal cell, as illustrated in the cartoon (C). Line styles represent different values of h (A) or p (B), as indicated. Default: $h = 100/a.u.$, $p = 3$.
